# Surface functionalization of aluminosilicate nanotubes with organic molecules

**DOI:** 10.3762/bjnano.3.10

**Published:** 2012-02-02

**Authors:** Wei Ma, Weng On Yah, Hideyuki Otsuka, Atsushi Takahara

**Affiliations:** 1 Institute for Materials Chemistry and Engineering, Kyushu University, 744 Motooka, Nishi-ku, Fukuoka 819-0395, Japan; 2 Graduate School of Engineering, Kyushu University, 744 Motooka, Nishi-ku, Fukuoka 819-0395, Japan; 3 International Research Center for Molecular Systems, Kyushu University, 744 Motooka, Nishi-ku, Fukuoka 819-0395, Japan

**Keywords:** chemisorption, imogolite, inorganic nanotube, surface functionalization.

## Abstract

The surface functionalization of inorganic nanostructures is an effective approach for enriching the potential applications of existing nanomaterials. Inorganic nanotubes attract great research interest due to their one-dimensional structure and reactive surfaces. In this review paper, recent developments in surface functionalization of an aluminosilicate nanotube, “imogolite”, are introduced. The functionalization processes are based on the robust affinity between phosphate groups of organic molecules and the aluminol (AlOH) surface of imogolite nanotubes. An aqueous modification process employing a water soluble ammonium salt of alkyl phosphate led to chemisorption of molecules on imogolite at the nanotube level. Polymer-chain-grafted imogolite nanotubes were prepared through surface-initiated polymerization. In addition, the assembly of conjugated molecules, 2-(5’’-hexyl-2,2’:5’,2’’-terthiophen-5-yl)ethylphosphonic acid (HT3P) and 2-(5’’-hexyl-2,2’:5’,2’’-terthiophen-5-yl)ethylphosphonic acid 1,1-dioxide (HT3OP), on the imogolite nanotube surface was achieved by introducing a phosphonic acid group to the corresponding molecules. The optical and photophysical properties of these conjugated-molecule-decorated imogolite nanotubes were characterized. Moreover, poly(3-hexylthiophene) (P3HT) chains were further hybridized with HT3P modified imogolite to form a nanofiber hybrid.

## Review

Surface functionalization of metal or metal-oxide surfaces has received considerable attention in recent years [1–3]. It presents an easy, accurate and precise approach for the fabrication of functional surfaces with highly controlled chemical properties. Functionalized surfaces can be used in a number of applications, including passivation of metal surfaces, adhesion promotion, and adsorption of biomolecules to substrates for sensing [1,4]. Recently, the assembly of organic molecules on inorganic nanostructures instead of flat surfaces has been demonstrated to be an effective process for preparing various previously untested functional organic/inorganic nanohybrids. The organic parts generally provide functional groups for the nanohybrids, while the inorganic parts act as the scaffold for organic molecules and determine both the individual morphology and the texture of the obtained nanohybrids [5–6]. Among various nanostructures with different shapes, nanotubes attract special research interest, not only because of their high mechanical strength, but also because of their large aspect ratios and ability to form network structures. It is no doubt that nanotubes with reactive surfaces and a reliable supply are preferred for the application as scaffold of organic molecules. Carbon nanotubes (CNTs) play an important role in the nanotube family. However, the surface of CNTs is inert for most molecules. On the contrary, clay nanotubes present a reactive surface for numerous coupling agents and are emerging as useful structural units for many kinds of nanohybrid materials [7–11].

For the assembly of organic molecules on an inorganic surface, most work has been carried out with alkyl silanes adsorbed on silicon oxide or with thiols adsorbed on noble metals [1,12–13]. A different class of self-assembling agents, namely phosphonic and phosphoric acids, has gained more and more attention due to their ability to bind to a wide range of metal-oxide surfaces [14]. Organosilane and organophosphorus coupling molecules show remarkably different reactivities. Silicon derivatives are prone to nucleophilic substitution, and the main reactions involved in the assembly process are hydrolysis and condensation reactions. Heterocondensation between the organosilanols and the inorganic part leads to the formation of Si–O–M bonds, while homocondensation between two coupling molecules leads to the formation of Si–O–Si bonds. The presence of a trace amount of water appears to be necessary for the formation of complete monolayers [15–16]. However, homocondensation increases as the water content increases and there is a risk of formation of multilayers due to the uncontrolled polymerization of the multifunctional organosilanes [17–18]. Phosphorus derivatives are much less sensitive to nucleophilic substitution than silicon derivatives are, because phosphorus has a higher electrophilicity compared to silicon. Consequently, P–O–C bonds are quite stable against hydrolysis, and P–O–H groups are quite stable against homocondensation. Thus, during the surface-modification process, they should form only monolayers, independent of the water content. Moreover, organophosphoric acids can selectively assemble on the surfaces of metal oxides rather than on SiO_2_ surfaces in an aqueous medium, due to the sensitivity of Si–O–P bonds to hydrolysis [19–21].

In this review paper, the chemisorption and assembly of several phosphonic-acid-containing organic compounds on imogolite nanotubes, based on the robust affinity between the phosphate groups and the nanotube surface, is reviewed.

### Aluminosilicate nanotube

#### Structure of imogolite

**Figure 1:**
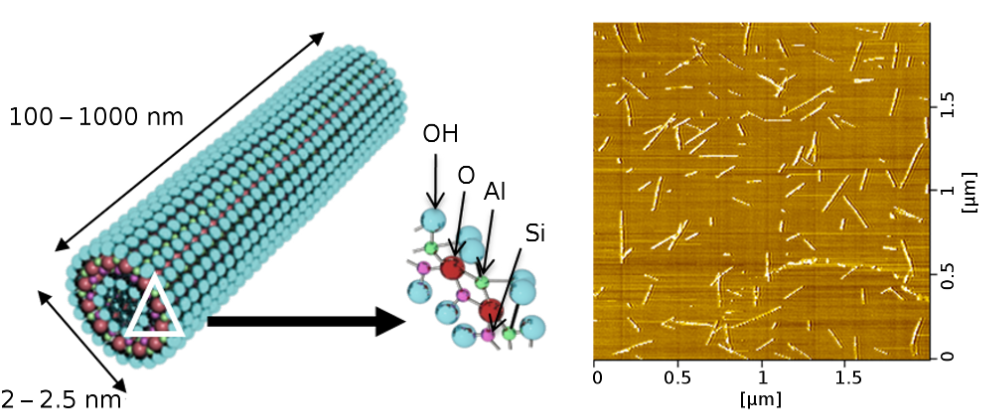
Schematic illustration of imogolite-nanotube structure (left). DFM image of imogolite (right).

**Figure 2:**
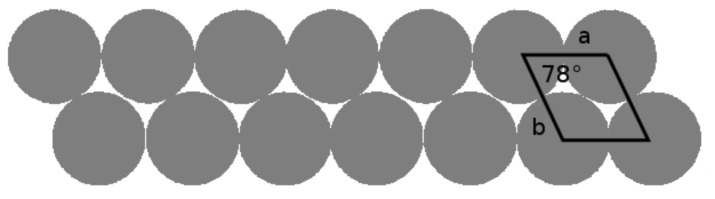
Monoclinic solid-state packing arrangement of the imogolite nanotubes.

Imogolite was discovered as early as 1962, and detail investigation using electron diffraction analysis by Cradwick et al. in 1972 confirmed its composition [Al_2_O_3_·SiO_2_·2H_2_O] [22]. The schematic representation of imogolite is shown in Figure 1. The gas adsorption data of N_2_, CO_2_, and CH_4_ concluded that imogolite possesses an inner-pore diameter of 1 nm [23]. The wall structure of imogolite comprises a layer of gibbsite on the outer wall, and a layer of silicate on the inner wall [22]. The latest crystallographic study showed that the imogolite tubes pack in a monoclinic arrangement through hydrogen bonds that form between the tubes (Figure 2) [24]. The artificial method to prepare imogolite was proposed by Farmer et al. in 1977 using the mild chemistry of Al(ClO_4_)_3_ and Si(OH)_4_ [25]. The formation mechanism of imogolite is not well understood, but an early study suggested that the evolution of the tubular morphology is started by the binding of isolated silicate groups to the gibbsite sheet, in which the tetravalent Si atoms pull the oxygen atoms of the gibbsite sheet into the curvature cylinder [26]. Attempts to tune the imogolite dimensions appear to be futile, as the tubular structure does not change significantly throughout the synthesis process and the formation of nanotubes occurs at an early stage [24]. Individually dispersing imogolite nanotubes on a transmission electron microscopy (TEM) carbon grid, by means of the droplet evaporation technique, has made semiquantitative analysis of imogolite dimensions possible [27]. Semiquantitative analysis on the TEM images supports the kinetic-growth mechanism, in which protoimogolites and short imogolites are observed in the initial stage of synthesis, and the average length of the nanotubes increases rapidly with reaction time [28].

#### Imogolite nanotubes through chemical synthesis and natural resource

The synthesis of imogolite was first reported by Farmer et al. in 1977 [25]. As a typical preparation method, a tetraethoxysilane solution mixed with aluminum chloride (AlCl_3_·6H_2_O) aqueous solution is pH adjusted to 5.0 giving the resulting solution of 2.4 mM of Al and 1.4 mM of Si. The pH adjusted solution is heated under reflux at 369 K for 120 h and gelated by NaCl solution at room temperature. The suspended material is washed with deionized water, filtered, and redispersed again in a weak acidic solution. Finally the imogolite solution is freeze dried and the final product of imogolite, which appears as cottonlike solid, is recovered. It is also possible to synthesize aluminogermanate imogolite, in which the Si is substituted with Ge, from a solution containing aluminum chloride and tetraethyl orthogermanate. The Ge substituted imogolite was found to be similar in tubular morphology to the natural imogolite and the external diameter could be expanded up to 3.3 nm by increasing the Ge/(Ge + Si) ratio. The expansion is attributed to the longer O–O distance in GeO_4_, which decreases the curvature of the gibbsite sheet [29–30].

**Figure 3:**
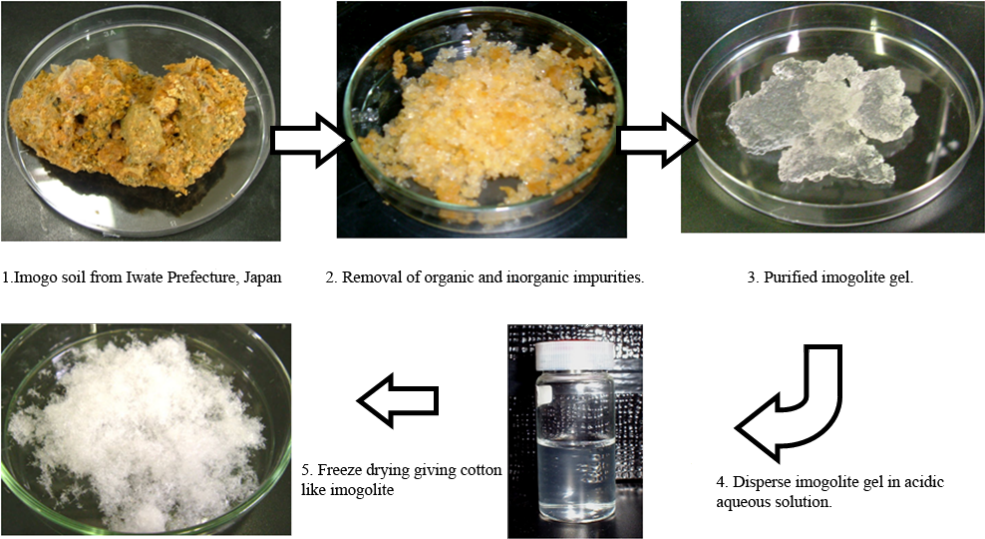
Purification steps of imogolite from imogo soil.

In an alternative method imogolite can be derived from glassy volcanic ash soil, but it then usually contains organic and inorganic impurities. These impurities can be separated from imogolite by purification as described in the literature [31]. In the typical purification procedure, the imogolite mineral collected from Kitakami, Iwate, Japan is suspended in water by ultrasonication. Occluded organic contaminants are removed by treating the mineral with hot 1.8 M H_2_O_2_, followed by citrate-bicarbonate (CB) to extract inorganic impurities (iron and manganese oxide). The resulting gel is washed with cold 0.5 M Na_2_CO_3_ to remove citrate remnants, and redispersed in weak acidic solution. The final product, cottonlike imogolite, is obtained by freeze-drying of the solution. Figure 3 shows the step-by-step purification procedure to recover the imogolite from the raw mineral.

### Surface functionalization of the imogolite-nanotube surface

#### Chemisorption of alkyl phosphate on imogolite nanotubes

**Figure 4:**
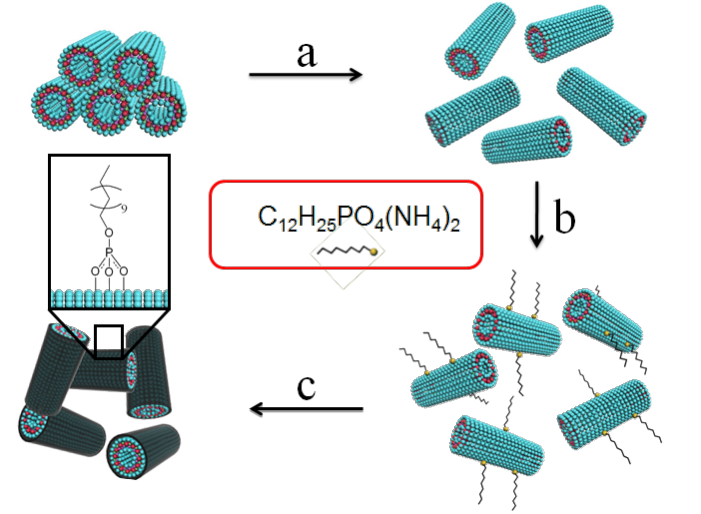
Schematic illustration of dodecylphosphate chemisorbing onto the surface of individually dispersed imogolite nanotubes: (a) Dispersion of freeze-dried imogolite powder into weak acidic water by electrostatic repulsion; (b) chemisorption of dodecylphosphate onto the outer surface of individually dispersed imogolite nanotubes; (c) purifying the product and solidifying it by freeze-drying. Reprinted with permission from W. Ma et al.*, Chem. Lett.* **2011,** *40,* 159–161 [33]. © 2011, The Chemical Society of Japan.

As mentioned above, imogolite is a very useful inorganic nanotube. The adsorption and assembly of organic molecules on the imogolite surface is expected to produce interesting results. Imogolite may act as a one-dimensional scaffold for functional molecules. Moreover, the surface energy of imogolite nanotubes can be lowered by the organic layer, and this can greatly improve the dispersibility of imogolite in organic solvents, as well as in various polymer matrices and nanocomposites. The metal–oxygen–phosphorus (M–O–P) interaction plays an important role for surface functionalization of imogolite nanotubes. The strong affinity between octadecylphosphonic acid and the imogolite surface has been reported by our group [32]. More recently, we developed an approach for anchoring alkyl chains on an imogolite surface from aqueous solution [33]. The adsorption of molecules on the inorganic surface from aqueous solution is particularly necessary for imogolite, because imogolite nanotubes are dispersible only in water, due to their AlOH surface. For this purpose, a step toward the modification of imogolite nanotubes at the nanotube level with alkyl phosphate from an aqueous solution was achieved by converting the water-insoluble alkyl phosphate into the corresponding water-soluble ammonium salt. The detailed assembly procedure is shown in Figure 4. The ammonium salt of dodecylphosphate (DDPO_4_(NH_4_)_2_) was precipitated from a 2-propanol solution of dodecylphosphoric acid (DDPO_4_H_2_) by the addition of ammonia. The outer surface of imogolite nanotubes is composed of aluminol groups, thus, it can be positively charged and dispersed under acidic conditions by electrostatic repulsion. It should be noted that surface modification of inorganic nanostructures in an aqueous solution is an environmentally friendly method.

**Figure 5:**
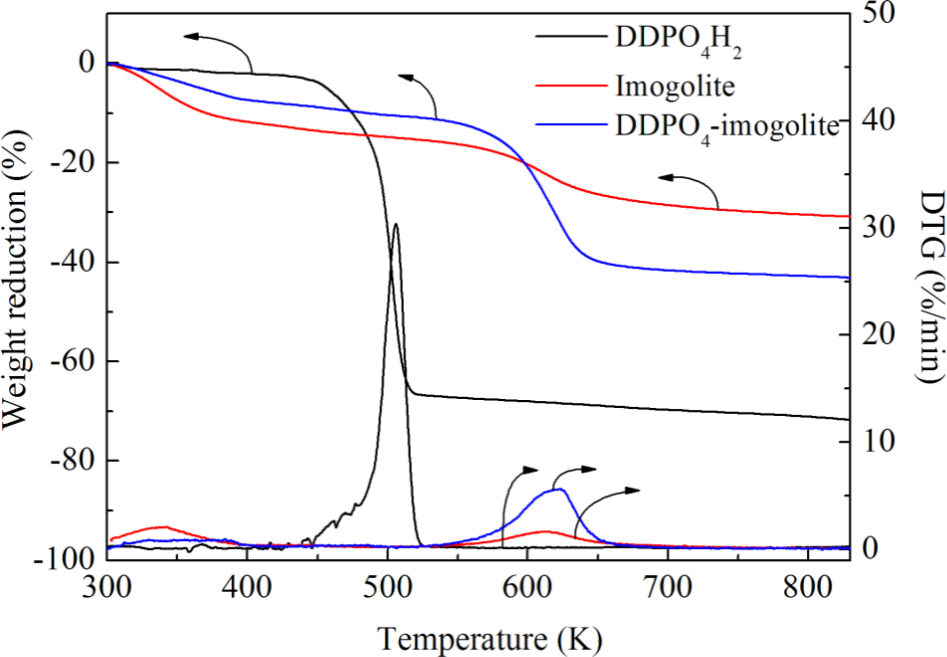
Thermogravimetric profiles of the original imogolite, DDPO_4_H_2_, and DDPO_4_-imogolite in N_2_ atmosphere at a heating rate of 10 K min^−1^.

Figure 5 shows the thermogravimetric profiles of the original imogolite, DDPO_4_-imogolite, and DDPO_4_H_2_. The synthetic imogolite loses 30% of its total mass in two steps. The first step is from 300 to 420 K with a weight loss of 13.5%, and the second step is from 420 to 800 K with a weight loss of 16.5%. The first step is attributed to the loss of adsorbed water, while the second one corresponds to the dehydroxylation of imogolite. DDPO_4_-imogolite has a similar weight-loss profile, although the second step also includes the decomposition of dodecylphosphate. The weight loss in the first step is 8.5%, while in the second step it is 33.5%. DDPO_4_H_2_ loses 70% of its initial mass at 800 K, indicating that phosphate groups are left over after the thermal decomposition. Thus, taking the weight loss of imogolite and dodecylphosphate into account, the imogolite content in DDPO_4_-imogolite is calculated to be 65.6%. Moreover, dodecyl phosphate exhibits an improved thermal stability in DDPO_4_-imogolite compared with the neat dodecylphosphoric acid. The onset decomposition temperature for DDPO_4_H_2_ is 420 K; while for the immobilized dodecyl phosphate the temperature increases to 520 K. This may be because, at the same temperature, the thermal motion of the anchored dodecylphosphate molecules is significantly restricted compared to the unanchored ones.

**Figure 6:**
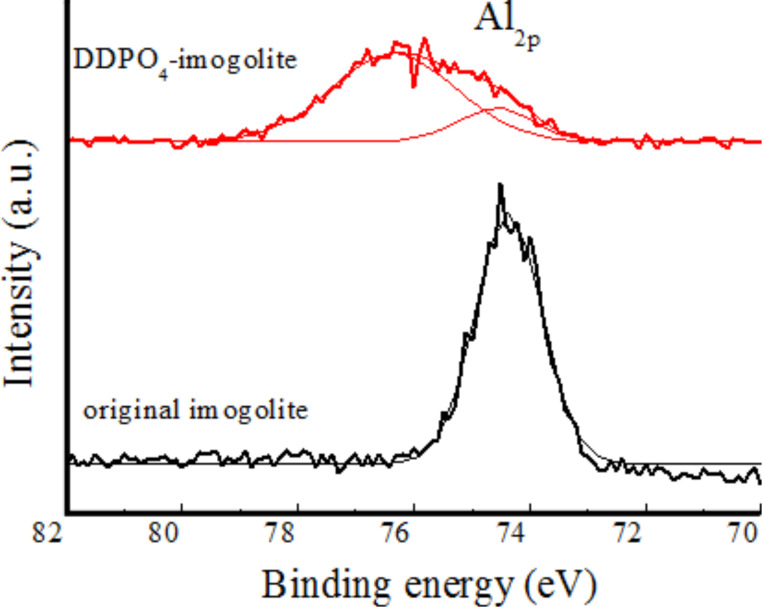
High-resolution XPS spectra for Al_2p_ of the original imogolite and DDPO_4_-imogolite. Adapted with permission from W. Ma et al., *Chem. Lett.* **2011, *** 40,* 159–161 [33]. © 2011, The Chemical Society of Japan.

The interaction between imogolite and DDPO_4_ is confirmed by IR measurements and X-ray photoelectron spectroscopy (XPS) [33]. The typical absorption bands of imogolite at 995, 935, and 560 cm^−1^ still exist, suggesting the retention of the Si–O–Al skeleton in imogolite nanotubes, while the absorption at 995 cm^−1^ is strengthened by the coexistent absorption of the phosphate groups. The absorption of P=O at 1239 cm^−1^ for DDPO_4_H_2_ and at 1201 cm^−1^ for DDPO_4_(NH_4_)_2_ disappears from the spectrum of DDPO_4_-imogolite, presumably due to the condensation between the phosphate groups and the aluminol groups. Figure 6 shows the high-resolution XPS spectra of Al_2p_. For the original imogolite, the Al_2p_ signal is found around 74.3 eV with a symmetric peak; while for DDPO_4_-imogolite the Al_2p_ peak becomes wide and asymmetric, and this can be fitted with two Gaussian curves corresponding to a contribution from peaks at 74.3 eV and 76.3 eV. The second component is ascribed to an increase in the positive charge on Al atoms due to the formation of Al–O–P bonds at the surface of imogolite, while the first indicates the unreacted Al–OH. Thus, it can be concluded that dodecylphosphate attaches to the surface of imogolite through covalent interaction.

**Figure 7:**
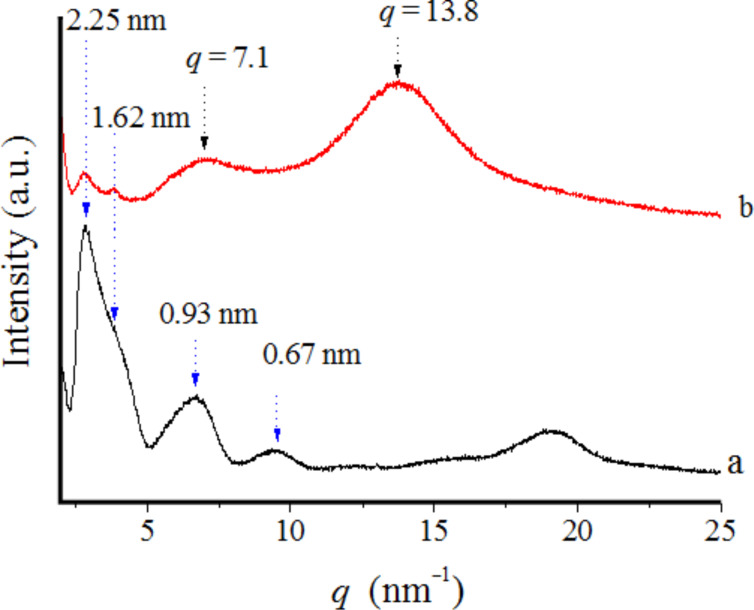
WAXD profiles of (a) original imogolite and (b) DDPO_4_-imogolite. Adapted with permission from W. Ma et al., *Chem. Lett.*** 2011,** *40,* 159–161 [33]. © 2011, The Chemical Society of Japan.

In order to obtain further insight into the molecular aggregation state of the DDPO_4_-imogolite, wide-angle X-ray diffraction (WAXD) measurements were carried out. Figure 7 shows WAXD profiles of the freeze-dried imogolite and DDPO_4_-imogolite. Scattering vector *q* [nm^−1^] is defined as *q* = (4π/λ)sinθ, where θ is the scattering angle. The *d*-spacing was calculated by *d* [nm] = 2π/*q*. The WAXD pattern of imogolite consists of a number of diffractions. The diffraction peaks at 2.25, 1.62, 0.93, and 0.67 nm for the freeze-dried imogolite can be assigned to the (100), (110), (001), and (211) planes of the quasi-monoclinic packing of the synthetic imogolite nanotubes [24]. For DDPO_4_-imogolite, the broad diffraction around *q* = 13.8 nm^−1^ is probably due to the disordered grafted alkyl chains. The diffractions at 2.25 and 1.62 nm suggest the presence of imogolite bundles. On the other hand, however, the intensity of the diffractions at 2.25 and 1.62 nm significantly decreased compared with those of the pure imogolite, indicating the exfoliation of the imogolite bundles. Imogolite cylinders may interact through their Al–OH groups, and bundles of imogolite tubes still exist even in weak acidic water. When dodecylphosphate attaches to the surface of these bundles, a one-dimensional core–shell structure forms with imogolite bundles as the core. However, it is expected that only tightly packed bundles can be maintained during the modification process. The modification agent may easily enter the gaps within the loosely packed bundles and adsorb on the surface.

**Figure 8:**
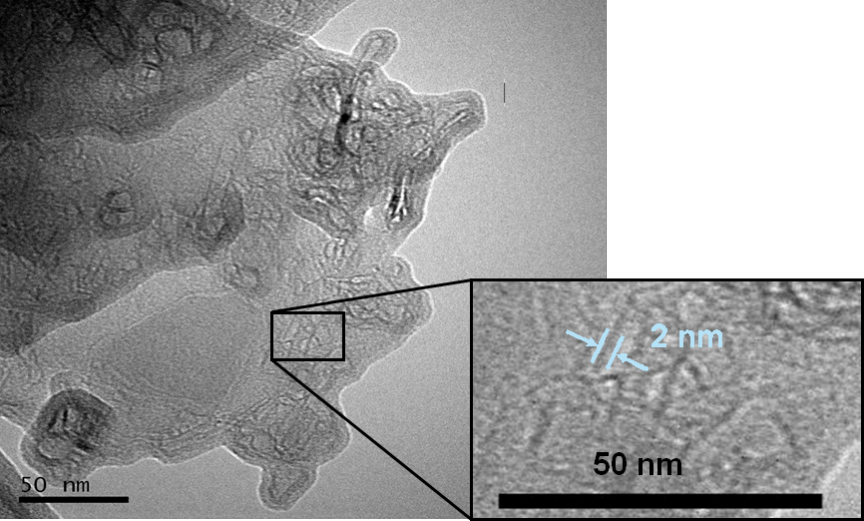
TEM image of DDPO_4_-imogolite. Reprinted with permission from W. Ma et al., *Chem. Lett.*** 2011,*** 40,* 159–161 [33]. © 2011, The Chemical Society of Japan.

The individual tubular structure of the dodecylphosphate modified imogolite is directly confirmed by TEM observation. The sample for TEM observation was prepared by placing a drop of the DDPO_4_-imogolite suspension (toluene as the solvent) on a carbon-coated copper grid and allowing it to dry in air. Figure 8 shows the TEM image of DDPO_4_-imogolite, in which fiberlike structures with a diameter about 2 nm were observed. This diameter is similar to that of the individual imogolite nanotubes, indicating that these are individual tubes rather than bundles. To the best of our knowledge, this is the first observation of individual imogolite nanotubes with a hydrophobic external surface.

**Figure 9:**
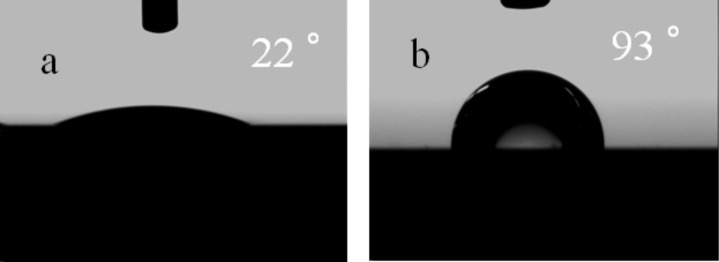
Static-contact-angle images of water droplets on a silicon wafer cast with (a) original imogolite and (b) DDPO_4_-imogolite. Reprinted with permission from W. Ma et al., *Chem. Lett.* **2011,*** 40,* 159–161 [33]. © 2011, The Chemical Society of Japan.

The wettability of this dodecylphosphate modified imogolite nanotube was evaluated by measurement of the water contact angle (CA) of the DDPO_4_-imogolite film. DDPO_4_-imogolite was dispersed in ethanol at a concentration of 2 mg mL^−1^, and this dispersion was cast onto a silicon wafer by spin coating. For comparison, an aqueous imogolite solution was also cast onto a silicon wafer by the same procedure. The static contact angle was measured by dropping 1 μL water onto the corresponding surface. As shown in Figure 9, the static contact angle for the original imogolite cast surface was 22°. In contrast, for the DDPO_4_-imogolite cast surface, the contact angle increased to 93°. This result indicates that the hydrophilicity of the external surface of imogolite is changed upon absorption of DDPO_4_, which converts the hydrophilic surface of imogolite nanotubes to a hydrophobic one.

#### Poly(methyl methacrylate) grafted imogolite nanotubes

The above content demonstrated the chemisorption of alkyl phosphonic chains on imogolite surface at the nanotube level from an aqueous solution. However, such low-molecular-weight compounds are insufficient to prevent nanotube aggregation. As a better alternative, the grafting of polymer chains from the nanostructure surface has been developed as a powerful technique for homogeneously dispersing nanostructures [34–36]. Several strategies can be used to graft polymers from the inorganic surface, including “grafting to”, “grafting through” and “grafting from” approaches [37–38]. In many cases, “grafting from” is preferred, in which the polymer chains are in situ grown from the surface by means of surface-initiated polymerization, and the grafting density is higher compared to the “grafting to” and “grafting through” approaches. The “grafting from” process can be performed with various polymerization techniques, from anionic and cationic to free-radical polymerization [39]. Free-radical polymerization is preferable to ionic processes on economic grounds, because it is easier to perform and much less sensitive to the presence of water.

**Figure 10:**
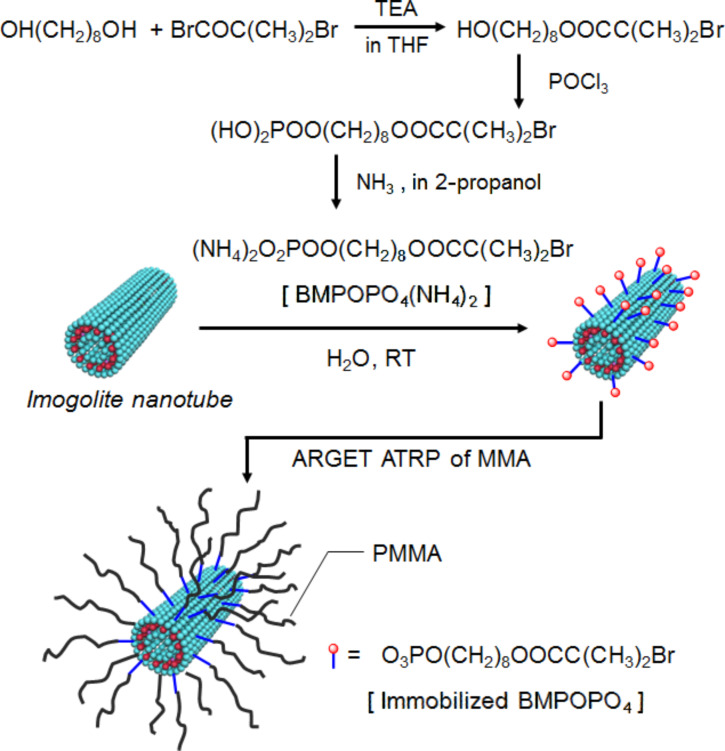
Schematic representation for the preparation of a PMMA grafted imogolite nanotubes. Reprinted with permission from W. Ma et al., *Chem. Commun.* **2011,** *47,* 5813–5815 [40]. © 2011, The Royal Society of Chemistry.

Recently, we reported the grafting of poly(methyl methacrylate) (PMMA) on the imogolite surface, in which surface-initiated radical polymerization, called “activators regenerated by electron transfer for atom transfer radical polymerization” (ARGET ATRP), was used [40]. ARGET ATRP is a newly developed controlled/living radical polymerization technique, and has been attracting more and more research interest due to its convenience, e.g., it can be carried out without strict deoxygenation and only needs ppm levels of catalyst [41–42]. Figure 10 presents the preparation procedure of a PMMA grafted imogolite nanotube.

To realize polymerization of MMA from the surface of imogolite nanotubes, a suitable surface-attachable ATRP initiator is needed. So far, various ATRP initiators that can be fixed on inorganic surfaces have been synthesized by several groups [43–47]. Among them, surface-attachable groups have almost exclusively been alkoxy- or chlorosilanes. However, organosilanes are not suitable for the modification of imogolite, because surface modification with organosilanes usually needs dry conditions in order to prevent unfavorable side reactions. Whereas with an AlOH functionalized external surface imogolite is a very hydrophilic material and can be dispersed only in acidic water by electrostatic repulsion [48]. Moreover, surface bonds between organosilane and the external surface of imogolite have been reported to be labile in a humid atmosphere [49]. On the other hand, organophosphorous compounds appear complementary to organosilanes, as they show an excellent affinity toward metal oxides [14,36,50]. In addition, they are rather insensitive to nucleophilic substitution and prone to heterocondensation (M–O–P bond formation) as compared to homocondensation (P–O–P). Thus, surface modification with organophosphorous compounds has the advantage of being operable in a wide range of solvents from aprotic to protic, and even in water.

**Figure 11:**
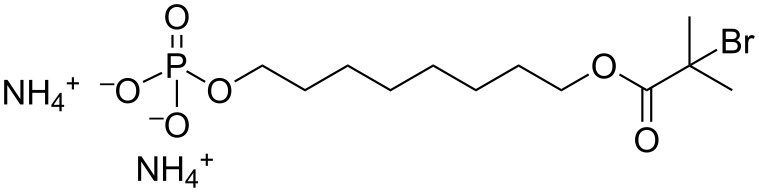
Chemical structure of BMPOPO_4_(NH_4_)_2_. Reprinted with permission from W. Ma et al., *Polymer* **2011,** *52,* 5543–5550 [53]. © 2011, Elsevier B.V.

In line with the above discussion, we synthesized an initiator carrying a phosphoric acid group, 8-(2-bromo-2-methylpropanoyloxy) octyl phosphoric acid (BMPOPO_4_H_2_), which was further converted to a water-soluble ammonium salt [BMPOPO_4_(NH_4_)_2_]. Figure 11 shows the chemical structure of this initiator molecule. To the best of our knowledge, the closest analogous molecules appear in two papers, in which 11-(2-bromoisobutyrate)-undecyl-1-phosphonic acid and its diethyl ester were synthesized [51–52]. However, these two molecules were both designed for application in organic solvents and are not soluble in water. The homogeneous modification of the imogolite surface can be achieved by using a water-soluble initiator carrying a surface-attachable group. In addition, the molecule we designed here seems capable of providing the modified imogolite with adequate hydrophobicity, as it contains a relatively long hydrophobic chain.

**Figure 12:**
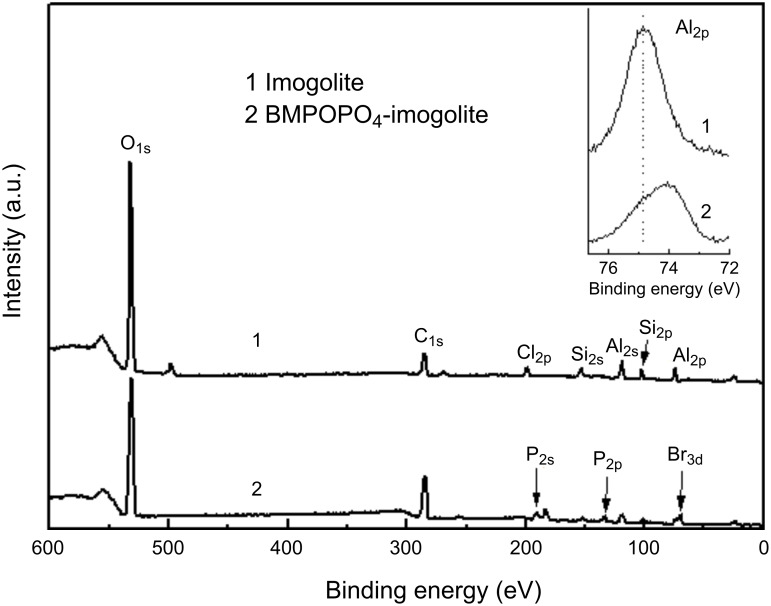
Wide-scan XPS spectra of the original imogolite and BMPOPO_4_-imogolite (inset, high-resolution XPS spectra of Al_2p_). Reprinted with permission from W. Ma et al., *Polymer* **2011,** *52,* 5543–5550 [53]. © 2011, Elsevier B.V.

The ATRP initiator BMPOPO_4_ was immobilized on the imogolite surface from an aqueous solution at room temperature. The pH is an important parameter in this modification reaction. A low pH value is favorable for the fine dispersion of imogolite in water. However, if the pH is too low, the phosphate group may cause the dissolution of metal oxides. It was reported that PhPO(OH)_2_ can cause the release of aluminum cations from the alumina surface by cleavage of Al–O–Al bonds at pH 4 [54]. In this work, the acidity of the initial reaction mixture was controlled and set to be pH 5 in order to avoid a similar dissolution process of imogolite, whose external surface is similar to that of alumina. In our case, a pH 5 acetate buffer was employed. The adsorption of BMPOPO_4_ onto the imogolite surface was confirmed by FT-IR measurements and XPS analysis. FT-IR spectra show that the Al–O–Si vibrations of imogolite at 992 and 930 cm^−1^ still exist after modification, indicating that this reaction does not destroy the structure of the imogolite nanotube [40]. The absorbance bands at 2932 cm^−1^ (C–H) and 1735 cm^−1^ (C=O) confirm the adsorption of the ATRP initiator onto the imogolite surface, while the absence of the N–H vibration band at 3130 cm^−1^ indicates that ammonium counter cations do not adsorb onto imogolite. XPS spectra (Figure 12) provide more information on the interaction between the imogolite surface and the ATRP initiator. In the wide-scan XPS spectra of BMPOPO_4_-imogolite, the characteristic peaks of P_2p_, P_2s_, and Br_3d_ were found at around 134.5, 191.8, and 70.0 eV, respectively. In addition, no signal of nitrogen was found, further confirming that the ammonium counter cations do not adsorb onto imogolite. The high-resolution XPS spectra of Al_2p_ show that the peak position of Al_2p_ shifts from 74.85 to 74.09 eV (Figure 12, inset) after modification, which is ascribed to a decrease in the positive charge on Al atoms because of the adsorption of the negatively charged phosphate groups. Thus, the initiator is attached onto the imogolite surface possibly through electrostatic adsorption. In this case, the electron density of the surface aluminum atoms becomes higher compared with the unmodified imogolite, due to the influence of the negatively charged phosphate groups. In contrast, the formation of Al–O–P covalent bonds has been reported to cause an increase in the positive charge on Al atoms, as mentioned above. The difference in bonding manner between DDPO_4_ and BMPOPO_4_ on the imogolite surface may be due to the different hydrophobicity of these two molecules. The amount of adsorbed BMPOPO_4_ was estimated to be 49 wt % by thermogravimetric analysis (TGA) [40].

The subsequent ARGET ATRP was carried out by using ascorbic acid (AA) as the reducing agent and anisole as the solvent. Ascorbic acid is insoluble in anisole, hence, the reduction of the Cu(II) complex takes place at the surface of solid ascorbic acid. The slow reaction rate of this heterogeneous redox process is beneficial for building up a necessary equilibrium between the activator (Cu(I) complex) and deactivator (Cu(II) complex). Polymeric products were isolated by precipitation from methanol. GPC data showed that grafted PMMA with molecular weights of *M*_n_ = 26600 and 32700, and corresponding molecular weight distributions of *M*_w_/*M*_n_ = 1.22 and 1.33 were obtained after a polymerization time of 50 and 90 min, respectively. Hence, grafted PMMA with controllable molecular weight can be achieved by controlling the reaction time.

**Figure 13:**
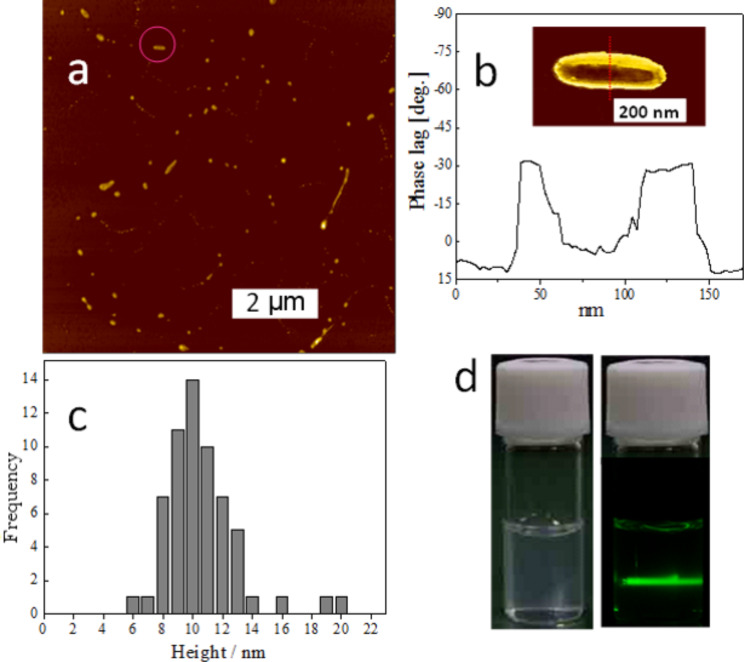
(a) A SFM height image of PMMA grafted imogolite (*M*_n_ = 32700, *M*_w_/*M*_n_ = 1.33). (b) A phase image (insert) and cross-sectional analysis of a PMMA-*g*-imogolite marked with a circle in (a). (c) Height distribution of the above sample as estimated by SFM. (d) Photographs of the THF dispersion of the same sample with a concentration of 10 mg mL^−1^ (the one on the right shows scattering of a green light beam by the dispersion due to the Tyndall effect). Reprinted with permission from W. Ma et al., *Chem. Commun.* **2011,** *47,* 5813–5815 [40]. © 2011, The Royal Society of Chemistry.

Bare imogolite cannot be dispersed in any organic solvent, but after modification with BMPOPO_4_, the resulting modified imogolite can be dispersed in various solvents. Unfortunately, the dispersions are neither homogenous nor stable. However, when PMMA was grafted to the surface of imogolite nanotubes, PMMA-*g*-imogolite showed good dispersibility in organic solvents, such as THF, chloroform, and toluene. As shown in Figure 13d the homogenous dispersion of PMMA-*g*-imogolite in THF with a concentration of 10 mg mL^−1^ was stable for more than two months.

Morphology of PMMA grafted imogolite nanotubes was observed with scanning force microscopy (SFM) in a dynamic force microscopy (DFM) mode employing a sharp diamondlike carbon (DLC) tip with a radius of curvature of 1 nm. Figure 13a shows a height image of one sample with *M*_n_ = 32700 and *M*_n_/*M*_w_ = 1.33. Discrete nanostructures were randomly distributed on the mica surface and no aggregation was observed, indicating excellent dispersibility of PMMA grafted imogolite. The high-resolution phase image and the corresponding cross-sectional analysis in Figure 13b indicates that PMMA grafted imogolite renders a hard middle part and a soft edge. This further confirms the core–shell structure of PMMA-*g*-imogolite. Figure 13c shows the height distribution of the above PMMA-*g*-imogolite analyzed from 60 SFM images. The average height value was determined to be 10.6 ± 2.5 nm, although there are still some images having heights of more than 15 nm. If we consider the sample with the smallest height value (6 nm) as containing an individual imogolite nanotube at the core, the ones with larger height values are expected to have nanotube bundles as their rigid cores.

**Figure 14:**
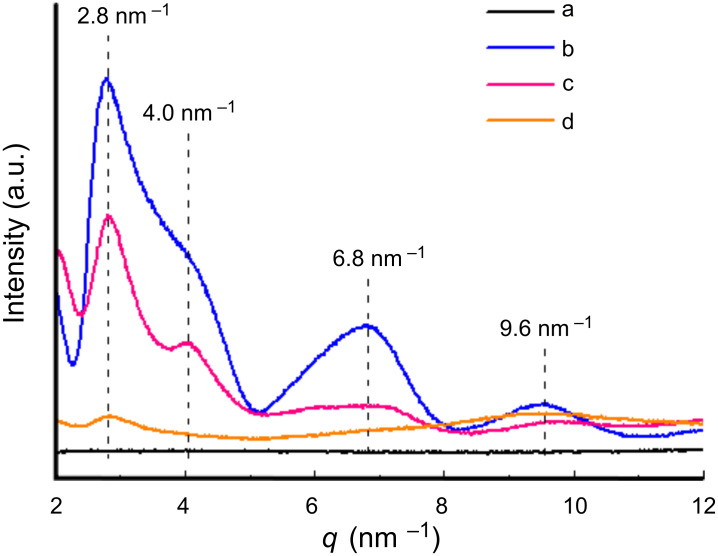
WAXD profiles of (a) quartz-glass capillary background, (b) bare imogolite, (c) BMPOPO_4_-imogolite, and (d) PMMA-*g*-imogolite with *M*_n_ = 32700 and *M*_w_/*M*_n_ = 1.33. Reprinted with permission from W. Ma et al., *Chem. Commun.* **2011,** *47,* 5813–5815 [40]. © 2011, The Royal Society of Chemistry.

Further evidence on the bundle structures was provided by wide-angle X-ray diffraction (WAXD) measurements. As shown in Figure 14, the diffraction peaks at *q* = 2.8, 4.0, 6.8, and 9.6 nm^−1^ can be assigned to the (100), (110), (001), and (211) planes of the parallel bundles of the imogolite nanotubes, respectively [24]. For BMPOPO_4_ modified imogolite, these four diffraction peaks still exist, suggesting the presence of imogolite bundles. In addition, the peaks at around *q* = 2.8 and 4.0 nm^−1^ become sharper than those of the bare imogolite and, as a result, can easily be distinguished from the overlapped profile, indicating the higher regularity of the bundles compared to that of the bare imogolite. Hence, it is reasonable to conclude that during the modification process only highly ordered imogolite bundles can remain. In addition, the diffraction from (001) plane at ca. *q* = 6.8 nm^−1^ becomes much weaker and broader, suggesting that the bundle size significantly decreased along the (001) plane direction. The above results indicate that small-sized imogolite bundles with high regularity form the rigid core of BMPOPO_4_-imogolite during the modification process. After surface-initiated polymerization of MMA, these small-sized imogolite bundles become the cores of PMMA-*g*-imogolite, and the diffraction of the (100) plane at around *q* = 2.8 nm^−1^ can still be observed. This result is consistent with the explanation for the SFM observation.

#### Terthiophene/imogolite hybrid

**Scheme 1:**
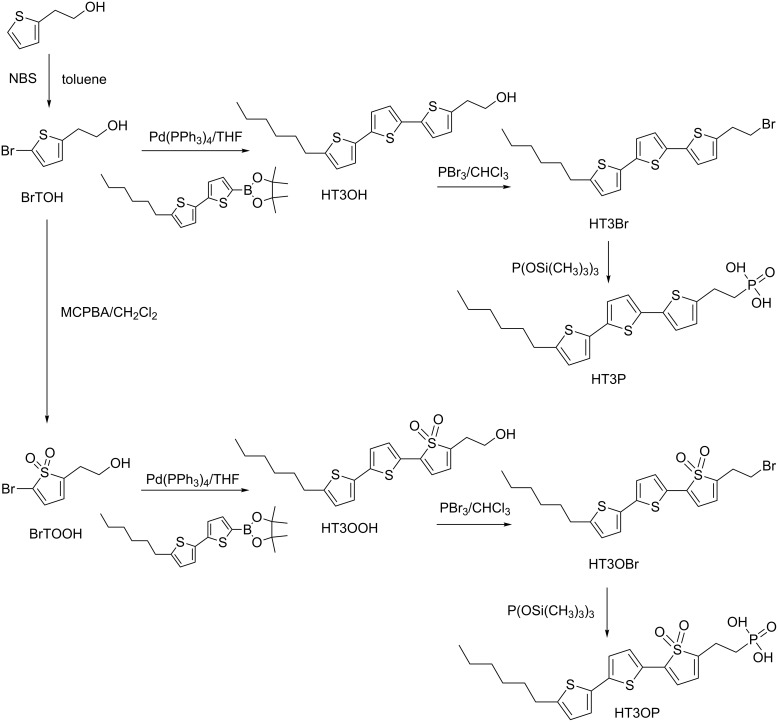
Synthesis pathway for electron donating (HT3P) and accepting (HT3OP) terthiophene of phosphonic acid derivatives. Reprinted with permission from W. O. Yah et al., *Bull. Chem. Soc. Jpn.* **2011,** *84,* 893–902 [60]. © 2011, The Chemical Society of Japan.

Grafting of functionalized molecules (porphyrins, phtalocyanines, viologens, rhodamine B, etc.) onto metal-oxide surfaces of SiO_2_, TiO_2_, ITO, WO_3_, and ZrO_2_ can induce the formation of well-defined nanoscopic photoactive molecular arrays of heterosupramolecular assemblies [55–56]. Imogolite lacks the intrinsic semiconductivity of the carbon nanotube, but it can be an interesting condensed phase for heterosupramolecular systems due to its high surface area for molecular component adsorption, abundance of empty surface sites for covalent binding of acidic anchoring groups, and high stability under ambient conditions. A more promising approach to render aluminosilicate nanotubes semiconducting is by functionalization with conjugated molecules, such as terthiophene with alkyl spacers consisting of –CH_2_CH_2_– and P=O(OH)_2_ (Scheme 1). Thiophene oligomers have been extensively studied in recent years due to their excellent optic properties. It has been reported that thiophene oligomers exhibit high quantum yields of photoluminescence, both in solution as well as the solid state, and a broad range of fluorescence frequencies in the entire UV–visible and the near-IR spectrum, through molecular engineering [57–59].

**Figure 15:**
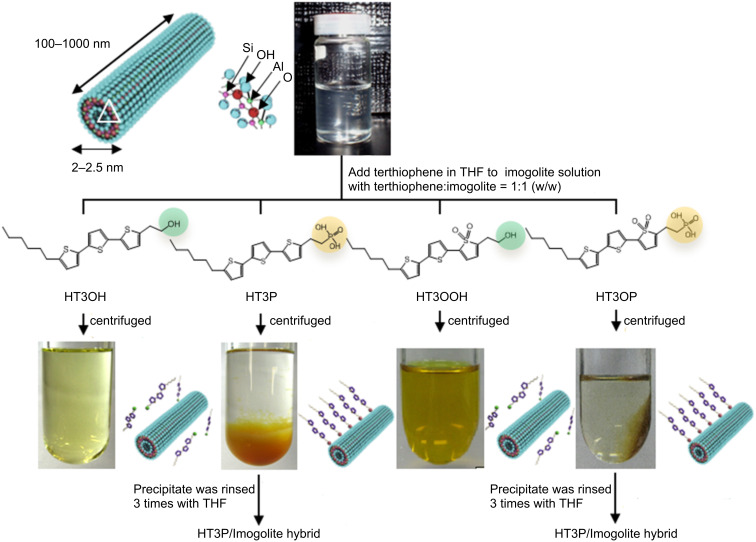
Schematic illustration of imogolite structure and the preparation of terthiophene/imogolite hybrid materials. Reprinted with permission from W. O. Yah et al., *Bull. Chem. Soc. Jpn.* **2011,** *84,* 893–902 [60]. © 2011, The Chemical Society of Japan.

For preparation of terthiophene/imogolite hybrid materials, imogolite solution was added dropwise to a THF solution of 2-(5’’-hexyl-2,2’:5’,2’’-terthiophen-5-yl)ethylphosphonic acid (HT3P) and stirred overnight at room temperature in the dark. The weight ratio of imogolite to HT3P was 1:1. HT3P/imogolite precipitate was obtained by centrifugation of the suspended solution and rinsing with fresh THF three times to remove weakly or nonchemisorbed HT3P. The precipitate was redispersed in deionized water before being freeze dried. Freeze drying of the precipitate resulted in a cottonlike yellow solid. The same preparation method was used for 2-(5’’-hexyl-2,2’:5’,2’’-terthiophen-5-yl)ethylphosphonic acid 1,1-dioxide (HT3OP) to produce the cottonlike pale brown solid of HT3OP/imogolite hybrid. As a control sample, no precipitate was observed for OH group derivatives (HT3OH and HT3OOH) (Figure 15).

**Figure 16:**
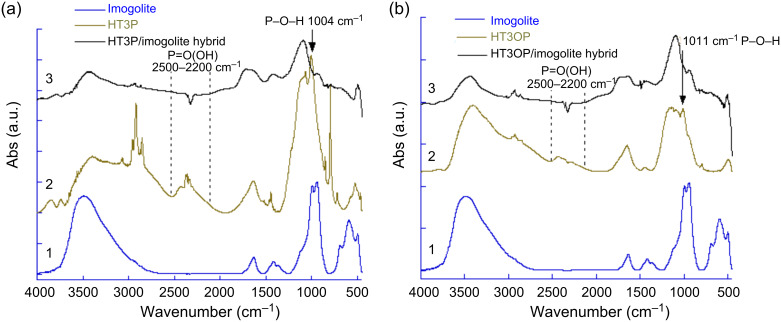
FTIR spectra (a) of HT3P/imogolite hybrid, HT3P, and imogolite, (b) of HT3OP/imogolite hybrid, HT3OP, and imogolite. Reprinted with permission from W. O. Yah et al., *Bull. Chem. Soc. Jpn.* **2011,** *84,* 893–902 [60]. © 2011, The Chemical Society of Japan.

In the FTIR spectrum of HT3P/imogolite hybrid in Figure 16a, the spectrum showed the characteristic absorptions corresponding to the CH_2_ stretching vibration of HT3P at 2850–2950 cm^–1^. The broadness of the peaks in the P–O region between 1200 and 900 cm^–1^ makes the result difficult to interpret, but the greatly diminished absorption at 2200–2500 cm^–1^ assigned to the OH stretching of the phosphonic acid groups indicates that the phosphonate headgroup strongly interacted with the imogolite surface [61]. In addition, the absence of the 1004 cm^–1^ band, which is assigned to P–O–H groups [62–63], was another indication that HT3P molecules were chemisorbed onto the surface of the imogolite nanofiber. The FTIR spectrum of HT3OP/imogolite hybrid as shown in Figure 16b gives a similar result with the disappearance of P–O–H at around 1011 cm^–1^ suggesting that HT3OP molecules also undergo chemisorptions when in contact with imogolite.

**Figure 17:**
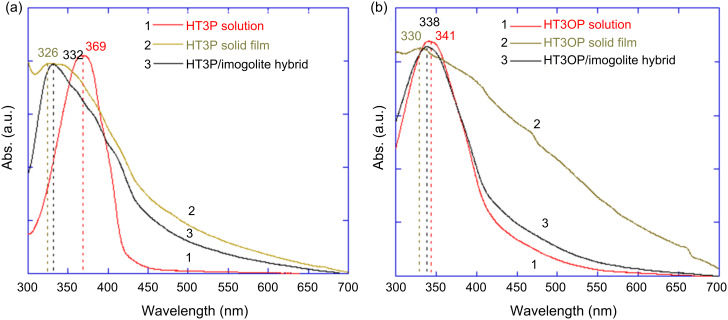
Normalized solid-state (cast film), imogolite hybrid and solution absorption spectra of (a) HT3P and (b) HT3OP. Reprinted with permission from W. O. Yah et al., *Bull. Chem. Soc. Jpn.* **2011,** *84,* 893–902 [60]. © 2011, The Chemical Society of Japan.

To investigate the optical properties of terthiophenes on the imogolite surface, a comparison of the UV–vis spectra of terthiophenes (HT3P and HT3OP)/imogolite hybrid between their solutions and solid-state counterparts was made (Figure 17). Blue-shifting was observed in the spectra of the solvent-cast film for both terthiophene hybrids to a different extent, and with a significantly broadened band. The spectral changes upon solidification from solution arise from two factors: Planarity and intermolecular interaction [64–66]. Normally, red-shifting occurs in the solid state when the molecular backbone is more planar compared to the isolated state in solution, due an increase in conjugation length. Thus, the blue-shifting observed in the spectra of thiophene/imogolite films compared to solution indicates that additional an intermolecular interaction was present that plays a role in controlling the solid-state optical properties [65].

**Figure 18:**
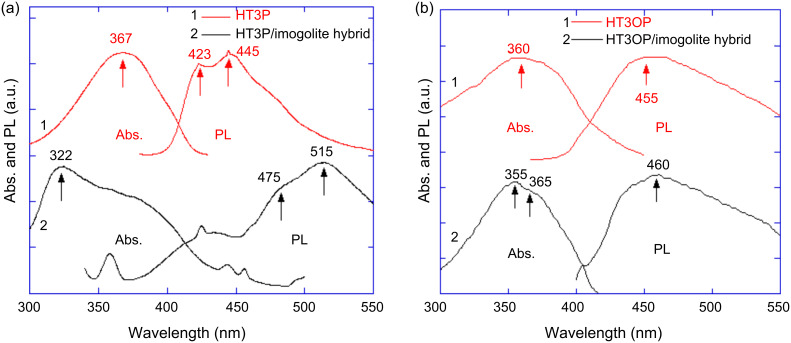
Fluorescence excitation/emission spectra of (a) HT3P, HT3P/imogolite hybrid and (b) HT3OP, HT3OP/imogolite hybrid. The emission wavelengths monitored for the excitation spectra and the excitation wavelengths used for the emission spectra were as follows: (a, 1) λ_em_ = 445 nm, λ_ex_ = 366 nm, (a, 2) λ_em_ = 326 nm, λ_ex_ = 519 nm. (b, 1) λ_em_ = 455 nm, λ_ex_ = 360 nm, (b, 2) λ_em_ = 460 nm, λ_ex_ = 460 nm. Reprinted with permission from W. O. Yah et al., *Bull. Chem. Soc. Jpn.* **2011,** *84,* 893–902 [60]. © 2011, The Chemical Society of Japan.

Fluorescence spectroscopy is a suitable analytical tool for monitoring the intermolecular interactions of terthiophene before and after chemisorption on imogolite. The absorption peak of the HT3P/imogolite hybrid at 322 nm, shifted from that of the HT3P in THF solution (λ_max_ 367 nm), provides additional proof for the formation of an H-type intermolecular interaction of terthiophene on the imogolite surface (Figure 18). The emission spectra also reveal the impact of intermolecular interaction in the hybrid with a peak at 515 nm that was red-shifted with respect to that of the HT3P solution (λ_max_ 445 nm) [67–68]. The formation of H-aggregates of HT3OP on the imogolite surface was also evidenced as the absorption peak shows blue-shifting relative to the HT3OP solution (λ_max_ 360 nm). The emission peak in the fluorescence spectrum of HT3OP/imogolite was red-shifted with respect to that of the HT3OP solution (λ_max_ 455 nm), suggesting the presence of an intermolecular interaction [67–68]. In the case of HT3OP/imogolite, however, the peaks are shifted to a lesser extent in the fluorescence spectrum compared to those of the HT3P/imogolite hybrid, due to distortion of the HT3OP backbone by S=O groups, which decrease its planarity and π–π interaction causing weaker H-aggregates of HT3OP on the imogolite.

**Figure 19:**
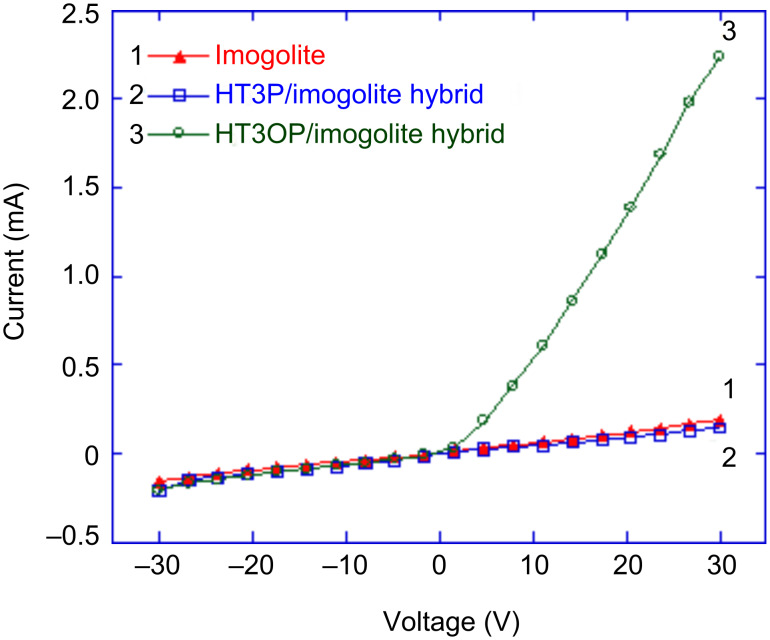
*I*–*V* curves of imogolite, HT3P/imogolite, and HT3OP/imogolite hybrid. Reprinted with permission from W. O. Yah et al., *Bull. Chem. Soc. Jpn.* **2011,** *84,* 893–902 [60]. © 2011, The Chemical Society of Japan.

Imogolite has been thought of as an insulator since imogolites consist of wide-bandgap alumina and silica. In fact, due to its unique tubular structure and high aspect ratio, imogolite can be used as an electron-emitting material and water sensor in nanoelectronic devices. The conductivities of the pure imogolite and of the terthiophene/imogolite hybrids were investigated by *I*–*V* measurement. By coating the pure imogolite or terthiophene/imogolite onto a silicon wafer and connecting by silver paste at two ends, the conductivity was measured by means of a source meter. The *I*–*V* plots, of current on the order of milliamperes versus applied bias voltage in the range from −30 to 30 V, are shown in Figure 19.

The averaged electrical conductance calculated from the forward bias region of the pure imogolite *I*–*V* curve is 5.9 µS. It was speculated that the current flow was due to charge hopping on the hydrated imogolite surface. Oh et al. studied the *I*–*V* characteristics of imogolite and proposed that bound water molecules contribute to the surface conductivity [69–70]. The current flow observed was attributed to the ability of OH groups on the imogolite surface to lose or gain positive charge (a proton) from water molecules resulting in a net change of surface charge. The electrical conductance of HT3OP/imogolite was improved to 60.8 µS, which is one order of magnitude greater than that of pure imogolite. It was observed that HT3OP/imogolite shows non-ohmic characteristics in the *I*–*V* curve, which signifies a disordered packing of HT3OP on the imogolite surface, in which it behaves like a semiconductor or metal–semiconductor Schottky junction. The HT3OP can act as an electron acceptor when interacting with imogolite, in which the high electron affinity of S=O of HT3OP causes a withdrawal of negative charge from imogolite resulting in the effective motion of positive charges on the imogolite surface. The introduction of HT3OP onto the imogolite surface amplifies the p-type conductivity of imogolite, which resembles the phenomenon of chemical doping of carbon nanotubes with alkaline metals [71–74]. On the other hand, the electrical conductance of HT3P/imogolite calculated from the forward bias region is 4.5 µS, which is lower than that before HT3P doping. Here, HT3P acts like an electron donor when interacting with imogolite. The p-type conductivity of imogolite is reduced when the HT3P thiophene ring transfers negative charge to imogolite, which restricts the effective motion of positively charged species on the imogolite surface.

#### Poly(3-hexyl thiophene)/imogolite nanofiber hybrid

**Figure 20:**
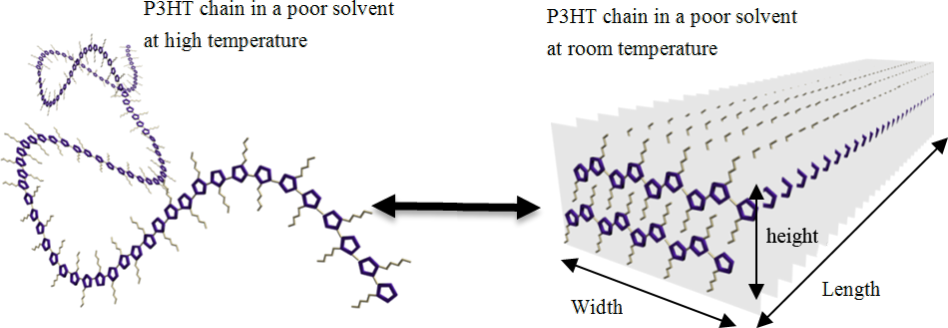
Schematic illustration of reversible formation of P3HT nanofiber.

Polythiophenes are one of the well-known families of conductive polymers, and their physicochemical properties, such as their synthesis, electrical and mechanical properties, thermochromism, solvatochromism, and crystal structure [75–78], have been extensively studied. Their optical properties, conductivity, and field-effect mobility (FEM) strongly depend on chain conformation and the solid-state-packing mode. For example, well-aligned and highly ordered crystalline polythiophene films will lead to significant improvements in conductivity and FEM; whereas the FEM of the disordered polythiophene film was below the detection level [79–80]. Generally, solidification of polythiophene by rapid evaporation of the polymeric solution in good solvent can result in a weak crystalline solid without perceptible morphological structure [81–82]. On the other hand, the slow cooling from 70 °C to room temperature of a P3HT solution in a poor solvent, such as xylene or anisole, leads to the formation of a fibrous semicrystalline structure (Figure 20). Nanofiber formation is always accompanied by a color change from orange to dark red, which is referred to as thermochromism [83].

**Figure 21:**
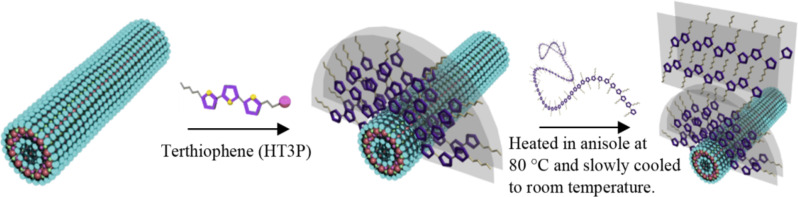
Fabrication and proposed molecular arrangement of P3HT/HT3P-imogolite nanofiber hybrid. Reprinted with permission from W. O. Yah et al., *J. Phys.: Conf. Ser.* **2011, ***272,* 012021 [86]. © IOP Publishing 2011.

Recently, a chemiresistive sensor based on a nanofiber hybrid of carbon nanotube/poly(3-hexylthiophene)- and carbon nanotube/hexafluoroiso-propanol-substituted polythiophene systems was reported [84]. Using a simple solution fabrication process, by dispersing carbon nanotubes in a polythiophene solution followed by spin coating of the solution onto a glass substrate, a highly sensitive and selective chemiresistor was successfully developed. Due to the favorable H-binding of the fluoro-alkyl groups of polythiophene to the phosphate ester, the nanofiber hybrid was reported to be able to detect several numbers of chemical warfare agents, such as dimethyl methylphosphonate (DMMP) and sarin gas [85]. Nevertheless, large-quantity synthesis of carbon nanotubes involves highly expensive and time-consuming preparation processes. Moreover, it is not practical for certain optical applications that use opaque carbon nanotubes, due to its conjugated π-system. Here, imogolite, the transparent hydrous aluminosilicate nanotube material, with its unique nanostructure was proposed as the inorganic nanotube to be hybridized with P3HT nanofibers. Reinforcement of P3HT nanofibers by imogolite is expected to impart additional mechanical and thermal stability to organic compounds, making the resulted hybrid material more durable under the outer environmental conditions. Therefore, it is crucial to develop a facile synthetic method capable of making uniform and template-free imogolite/P3HT nanofiber hybrids in bulk quantities. Such a synthetic method would be useful for tuning the properties of sensors and photovoltaic or light-emitting devices, which are dependent on well-defined low-dimensional structures. In order to improve compatibility with the P3HT nanofibers, hydrophilic Al–OH groups on the imogolite surface were modified with alkyl phosphonic acid substituted terthiophene (HT3P), as shown in Figure 21. The molecular aggregation states and molecular orientation of the P3HT chain on imogolite were investigated.

**Figure 22:**
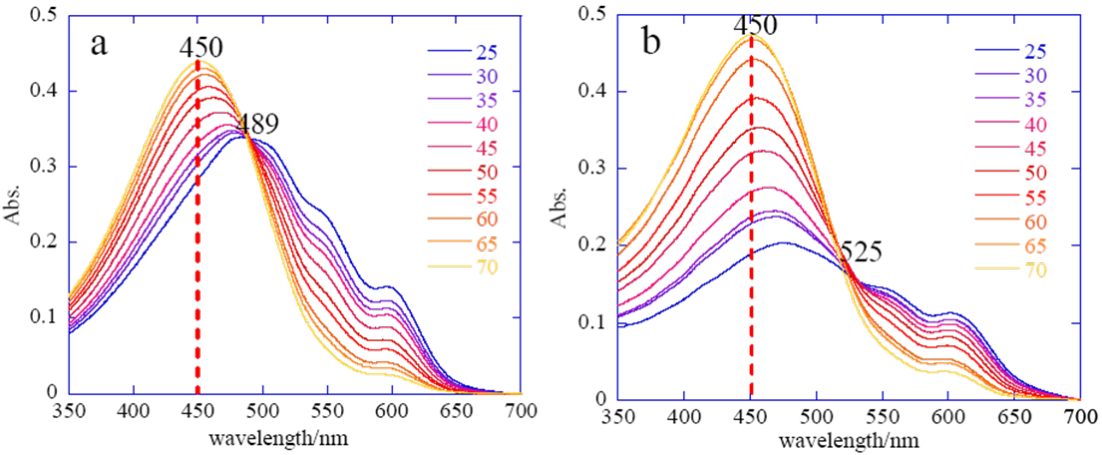
UV–vis absorption spectra of P3HT (a) and P3HT/HT3P-imogolite hybrid (b) in anisole (0.0005%) during cooling. Reprinted with permission from W. O. Yah et al., *J. Phys.: Conf. Ser.* **2011, ***272,* 012021 [86]. © IOP Publishing 2011.

Figure 22 displays the evolution of the absorption spectra of the P3HT solution (a) and the P3HT/HT3P-imogolite nanofiber hybrid (b) cooled from 70 °C to 20 °C. Notably, both spectra exhibit a single peak with λ_max_ of 450 nm at 70 °C. The spectrum resembles that observed for the P3HT/chloroform system indicating that P3HT was completely dissolved in anisole at the higher temperature. When cooled to room temperature, the band intensity at λ_max_ = 450 nm decreases in both spectra but is compensated by the appearance of vibronic structure at longer wavelengths (500–650 nm). The isosbectic points observed at 489 nm and 525 nm indicate that P3HT and P3HT/HT3P-imogolite exhibit both isolated coil-like conformation and rodlike conformation in the solution [87]. The isosbectic point of P3HT/HT3P-imogolite is slightly shifted to a longer wavelength compared to P3HT and was ascribed to a larger amount of the P3HT/HT3P-imogolite aggregate. Upon hybridization, HT3P-imogolite greatly restricts the rotational motion of the P3HT backbone, such that it produces a much longer conjugation length than pure P3HT.

**Figure 23:**
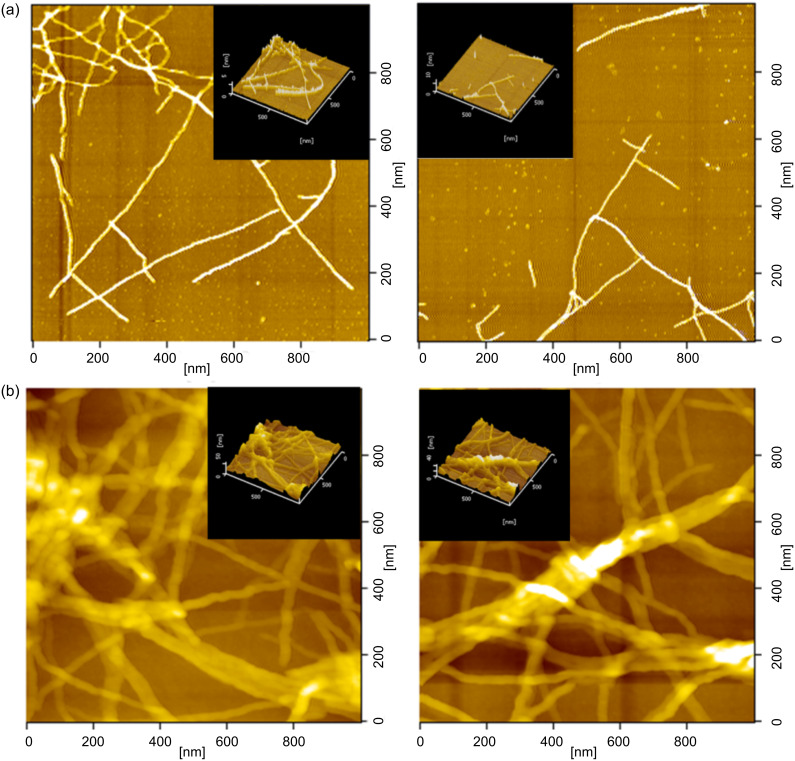
DFM images of (a) P3HT nanofiber and (b) P3HT/HT3P-imogolite nanofiber hybrid. Adapted with permission from W. O. Yah et al., *J. Phys.: Conf. Ser.* **2011, ***272,* 012021 [86]. © IOP Publishing 2011.

Dynamic force microscopy (DFM) has proved to be a powerful tool for the direct observation of the aggregation of polymeric nanofibers. By spin coating a dilute solution in anisole, a network of nanofibers more or less entirely covers the silicon substrate. The dimensions of the nanofiber were determined from DFM images; as shown in Figure 23a, P3HT nanofibers have a width and a length on the order of ca. 15 nm and 1 μm, respectively. The thickness, as estimated from the height image of DFM, was on the order of ca. 5 nm. On the other hand, it was observed that the heights of the nanofiber hybrids were 2 to 3 times larger than that of the P3HT nanofiber, indicating the formation of the bundle of the imogolite nanofiber [22]. Judging from the DFM images, the nanofiber hybrid as shown in Figure 23b exhibited a swollen morphology compared to the pure P3HT nanofiber. It was speculated that the swollen morphology was caused by the intertwining effect of the P3HT nanofiber with the imogolite bundle.

**Figure 24:**
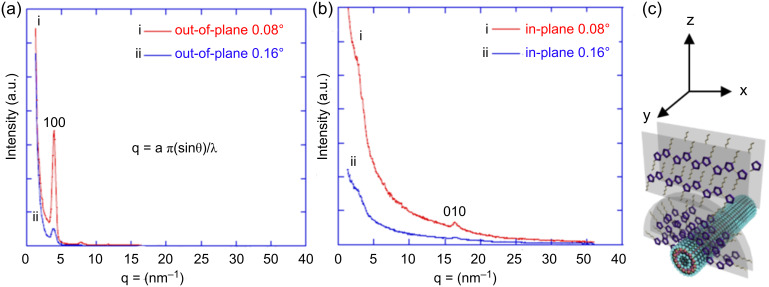
Out-of-plane (a) and in-plane (b) GIWAXD patterns of P3HT/HT3P-imogolite nanofiber hybrid. (c) Schematic illustration of the molecular arrangement of P3HT on imogolite. Reprinted with permission from W. O. Yah et al., *J. Phys.: Conf. Ser.* **2011, ***272,* 012021 [86]. © IOP Publishing 2011.

Recent studies on P3HT by grazing-incidence X-ray diffraction revealed the crystallinity and nanostructure in the nanofiber. The structure of P3HT nanofibers is similar to crystalline microdomains in which the P3HT chains pack in lamellar sheets perpendicular to the nanofiber axis. The orientation of the P3HT crystalline phase on the imogolite surface was studied by grazing-incidence wide-angle X-ray diffraction (GIWAXD), as shown in Figure 24. The out-of-plane GIWAXD pattern revealed only those crystalline plane positions that are in a direction parallel to the *x*–*y* axis. Likewise, in the in-plane GIWAXD pattern, only crystalline planes aligned to the *z*-axis are revealed.

In the out-of-plane GIWAXD pattern, the peaks at *d* = 4 (100) and 7.8 nm (200) were attributed to the ordering of the P3HT hexyl side chains. In other words, the nanofiber height corresponds to the stacking of the hexyl side chains and was parallel to the *z*-axis of the unit cell. The intensity at (100) was dramatically reduced when the incidence angle, α_i_ = 0.08°, was increased to 0.16°, suggesting that the semicrystalline P3HT mostly resides on the outermost region of the nanofiber hybrid. For the in-plane GIWAXD pattern, one noticeable diffraction peak associated with the (010) diffraction was observed. The diffraction peak corresponds to the π–π* stacking of the P3HT thiophene ring and was parallel to the *y*-axis, i.e., the nanofiber direction. Again, the (010) peak was diminished when the incidence angle, α_i_ = 0.08°, was increased to 0.16°. These results indicate that P3HT chains reside on the outermost region of nanofiber hybrid along the imogolite axis.

## Conclusion

This paper reviews the recent progress in the surface functionalization of imogolite nanotubes, which is based on the robust affinity between the phosphate group of the organic molecule and the aluminol (AlOH) surface of the imogolite nanotube. Surface modification of imogolite at the nanotube level is achieved from an aqueous solution by using a water-soluble ammonium salt of an alkyl phosphate. In addition, poly(methyl methacrylate) (PMMA) grafted imogolite nanotubes are prepared through a surface-initiated polymerization. PMMA grafted imogolite nanotubes can be homogenously dispersed in various organic solvents. A water-soluble surface-attachable ATRP initiator, BMPOPO_4_(NH_4_)_2_, contributes to the successful polymer-grafting process. Furthermore, the assembly of conjugated molecules, HT3P and HT3OP, on the imogolite nanotube surface was described. UV–vis spectra indicate that both HT3P and HT3OP exhibit an H-aggregate formation on the imogolite surface. An increase in the conductivity of imogolite is detected when assembled with electron-withdrawing HT3OP molecules. Further hybridization of HT3P assembled imogolite with P3HT, using a poor solvent, results in a P3HT/HT3P-imogolite nanofiber hybrid. UV–vis, DFM and GIWAXD studies showed that a P3HT nanofiber wrapping around the HT3P-imogolite nanotube causes the increase in diameter of the resultant nanofiber hybrid. It is believed that surface functionalization of the imogolite nanotube is an effective way to obtain nanomaterials with practical applicability.
